# Mortality Trends in Colorectal Cancer in China During 2000–2015: A Joinpoint Regression and Age-Period-Cohort Analysis

**DOI:** 10.5888/pcd15.180329

**Published:** 2018-12-13

**Authors:** Wenyuan Yu, Junfeng Jiang, Lingling Xie, Baojing Li, Huijuan Luo, Yuanshan Fu, Shu Chen, Chenkai Wu, Hao Xiang, Shenglan Tang

**Affiliations:** 1 Department of Global Health, School of Health Sciences, Wuhan University, Wuhan, China; 2 Global Health Institute, Wuhan University, Wuhan, China; 3 Global Health Research Center, Duke Kunshan University, Kunshan, China; 4 Duke Global Health Institute, Duke University, Durham, North Carolina

## Abstract

**Introduction:**

As lifestyles have increasingly become westernized in China, public health strategies have increasingly focused on cancer prevention. The objective of this study was to describe trends in colorectal cancer (CRC) mortality and the age, period, and cohort effects of CRC mortality in urban and rural China from 2000 to 2015.

**Methods:**

We collected CRC mortality data from the *China Health Statistics Yearbook*. We used joinpoint regression analysis to estimate the slope of mortality trends. We then used the age-period-cohort (APC) model with intrinsic estimator to estimate the age, period, and cohort effects of CRC mortality.

**Results:**

CRC mortality was higher in urban areas than in rural areas, and the average annual percentage change was also larger in urban areas (4.1%) than in rural areas (3.7%). CRC mortality risk was higher among older adults than among adults aged 20 to 24: the relative risk among adults aged 60 to 64 was 31.09 times higher in urban China and 11.46 times higher in rural China. CRC mortality risk increased with period: compared with period 2000, the relative risk was 1.01 in period 2005, 1.36 in period 2010, and 1.42 in period 2015 in urban China and 1.12 in period 2005, 1.24 in period 2010, and 1.69 in period 2015 in rural China. More recent cohorts had lower CRC mortality risk: compared with the cohort born during 1920–1924, the relative risk of cohort 1950–1954 was 0.70 in urban China and 0.69 in rural China.

**Conclusion:**

More interventions to reduce the burden of CRC should be conducted, and it is more necessary for older people and urban residents to adopt a healthy lifestyle in China.

## Introduction

Trends in colorectal cancer (CRC) mortality vary by country. During the last 2 decades, CRC mortality declined sharply in developed countries such as the United States and Canada (1,2). However, trends in CRC mortality in undeveloped countries usually show a shift from fluctuation to a gradual rise (3). For example, the annual percentage change in CRC mortality was 5.7% among men and 6.1% among women in the Philippines from 2001 to 2010 and 2.5% among men and 1.8% among women in Brazil from 1993 to 2004 (3).

CRC mortality may vary with age (age effect), period (period effect), and cohort (cohort effect). Age effect is the variation within a chronological age group (4), period effect is the variation during a period or calendar year that influences all age groups simultaneously (2,4), and cohort effect is attributed to distinct life courses of people born in different years (5). The diet of Chinese residents has changed to a high-fat, low-fiber diet, which has made obesity an increasingly severe problem (5,6). At the same time, however, medical conditions in China have improved and public health strategies have been progressively emphasized (7–9). In 2003, the Ministry of Health of China issued a governmental outline of the China Cancer Prevention and Control Program (2004–2010). This outline defined CRC as 1 of 8 key malignancies requiring intensive prevention and control. In 2012, China implemented the Cancer Early Detection and Treatment Project. The project, which was implemented in urban areas but not rural areas, included CRC as 1 of 5 targeted malignancies (10,11). In summary, changes in lifestyles have affected CRC mortality in China and more attention has been paid to CRC prevention, but disparities in medical conditions and prevention strategies still exist between urban and rural China.

A study by Wang et al showed the net period effect of total cancer mortality risk increased by 35.70% from period 1990 to period 2010, and cancer mortality risk consistently decreased with later-born cohorts in rural China (12). Another study showed that although CRC mortality decreased with period, it increased with cohort during 1971–2010 in Taiwan (13). No study has analyzed trends in CRC mortality in Mainland China, let alone differentiating age, period, and cohort effects.

The objective of our study was to estimate the age, period, and cohort effects of CRC mortality and compare the effects between urban and rural areas. Findings of our study would lay a foundation for discussing changes in various aspects of CRC mortality and assist in examining CRC risk factors in a temporal and historical context.

## Methods

The *International Classification of Diseases, 10th Revision* (14), defines CRC as malignant neoplasm of the colon (C18) and malignant neoplasm of the rectum, rectosigmoid junction, and anus (C19–21).

We collected CRC mortality data in urban and rural China for 2002 through 2015 from the *China Health Statistics Yearbook* (15). Data for 2000 were not available; as a substitute we used the available data from the nearest year (2002) in several analyses. The *China Health Statistics Yearbook* is an annual publication that comprehensively describes the health status of China; it covers 16 aspects, such as medical institutions, health expenditures, and causes of death and injury. Data in the *China Health Statistics Yearbook* are collected primarily from disease surveillance systems, such as the Infectious Disease Reporting System. We collected CRC mortality data from the Resident Death Surveillance System; these data are population-based data and are representative of the whole Chinese population (15).

### Joinpoint analysis

We used joinpoint regression analysis to assess the slope of CRC mortality trends in urban and rural China during 2000–2015. For this analysis, we substituted 2002 data for 2000 data. We fitted the joinpoint regression on a log scale because CRC mortality data followed a Poisson distribution. Joinpoint regression analysis fitted a series of connected lines to CRC mortality. We joined straight-line segments at joinpoints, where the slope of mortality trend significantly changed. Once we identified the line segments, we calculated annual percentage change to describe the temporal trend of CRC mortality (16). We used Joinpoint software version 4.5.0.1 (https://surveillance.cancer.gov/joinpoint/) to run the joinpoint regression. We also calculated the average annual percentage change to describe the overall trend. All tests were 2-sided with a significance level of .05.

### Age-period-cohort model with intrinsic estimator

We developed an age-period-cohort (APC) model to distinguish 3 temporal variations in the phenomena of interest: age, period, and cohort effects. Generally, the APC model can be expressed as


*Y_j_* = μ + α × Age*_j_* + β × Period*_j_* + γ × Cohort*_j_* + ε*_i_*

where *Y*_j_ denotes the outcome, CRC mortality; α, β and γ denote the estimated coefficients of age, period, and cohort for group *j*, respectively; μ denotes the intercept, and ε_i_ denotes the residual (17).

There is, however, an exact linear relationship among age, period, and cohort, so it is impossible to estimate the net effects of age, period, and cohort directly. This inability is called the identification problem. Our study used the APC model with intrinsic estimator, which has been confirmed to be unbiased, valid, and asymptotic in solving the identification problem (17).

We used intrinsic estimator to analyze the data collected from the *China Health Statistics Yearbook*. We needed consecutive 5-year data for intrinsic estimator; for this analysis, we substituted 2002 data for 2000 data. We focused on adults aged 20 to 84, and we classified data into 13 age groups (from 20–24 to 80–84), 19 birth cohort groups (from 1920–1924 to 1995-1999), and 4 periods (2000, 2005, 2010, and 2015). We excluded people younger than 20 years, because 1) CRC mortality data for this age group were not complete, 2) CRC mortality for this age group is very low, 3) and we found no change in CRC mortality for people aged younger than 20 from 2000 to 2015. We also excluded adults older than 85, because adults older than 85 were recorded as 1 group in the *China Health Statistics Yearbook* and these data could not be analyzed in intrinsic estimator.

The goodness of fit of the APC model was evaluated by using 3 indexes: fitting deviance, Akaike Information Criterion (AIC), and Bayesian Information Criterion (BIC). To interpret the age, period, and cohort effects more intuitively, we transformed the coefficients into relative risk. The transformation was expressed as 

RR*_ai_* = e^Coef^
*^ai^*
^-Coef^
*^a1^*

where RR*_ai_* denoted the relative risk of age (or period or cohort) of group *i*, Coef*_ai_* denoted coefficient of age (or period or cohort) of group *i*, Coef*_a1_* denoted coefficient of the reference age group (or period or cohort group). We chose each first group as the reference: first age group (age group 20–24), first period (2000), and first cohort group (1920–1924).

We used Stata version 12.0 (Stata Corp, LLP) to run intrinsic estimator.

## Results

**Figure 1:**
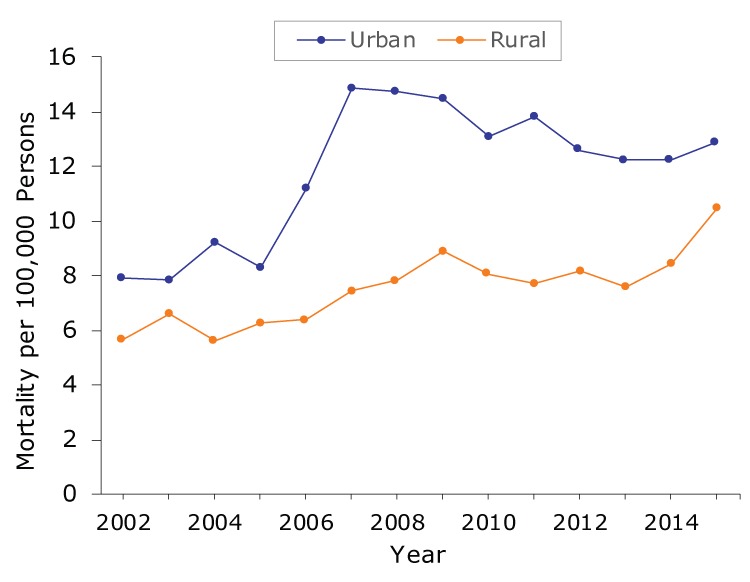
Colorectal cancer mortality per 100,000 persons in urban and rural China, 2002–2015. Data source: *China Health Statistics Yearbook* (15).

We found 2 trends in CRC mortality in urban China and 1 trend in rural China (Table 1). Overall, in urban areas, CRC mortality increased from 2002 to 2015 by an average of 4.1% per year. CRC mortality increased significantly in urban areas during 2002–2008; mortality increased from 7.90 per 100,000 persons in 2002 to 14.71 per 100,000 persons in 2008 (Figure 1), and the annual percentage change from 2002 to 2008 was 12.3% (Table 1). However, during 2008–2015, CRC mortality decreased in urban areas, albeit not significantly, from 14.71 per 100,000 persons in 2008 to 12.92 per 100,000 in 2015. In contrast, CRC mortality increased significantly in rural areas; mortality increased from 5.69 per 100,000 persons in 2002 to 10.46 per 100,000 persons in 2015 (Figure 1), and the average annual percentage change from 2002 to 2015 was 3.7% (Table 1).

**Table 1:** Trends in Colorectal Cancer Mortality in Urban and Rural China, 2000–2015^a^

Area	Trend	Years^b^	Average Annual Percentage Change (95% CI) [*P* Value^c^]	Average Annual Percentage Change 2000–2015 (95% CI) [*P* Value^c^]
Urban	Trend 1	2000–2008	12.3 (6.6 to 18.4) [<.001]	4.1 (1.2 to 7.1) [.005]
	Trend 2	2008–2015	−2.4 (−6.4 to 1.7) [.16]	
Rural	Trend 1	2000–2015	3.7 (2.3 to 5.1) [<.001]	3.7 (2.3 to 5.1) [<.001]

Abbreviation: CI, confidence interval.

a Data source: *China Health Statistics Yearbook* (15).

b Data for 2000 were not available; thus, we used as a substitute the available data from the nearest year (2002).

c Different from 0 at α = .05; determined by *t* test.

**Table:** 

Year	Urban	Rural
2002	7.90	5.69
2003	7.87	6.57
2004	9.23	5.6
2005	8.31	6.3
2006	11.17	6.4
2007	14.9	7.42
2008	14.71	7.84
2009	14.45	8.9
2010	13.12	8.07
2011	13.79	7.74
2012	12.59	8.18
2013	12.27	7.57
2014	12.24	8.43
2015	12.92	10.46

**Figure 2:**
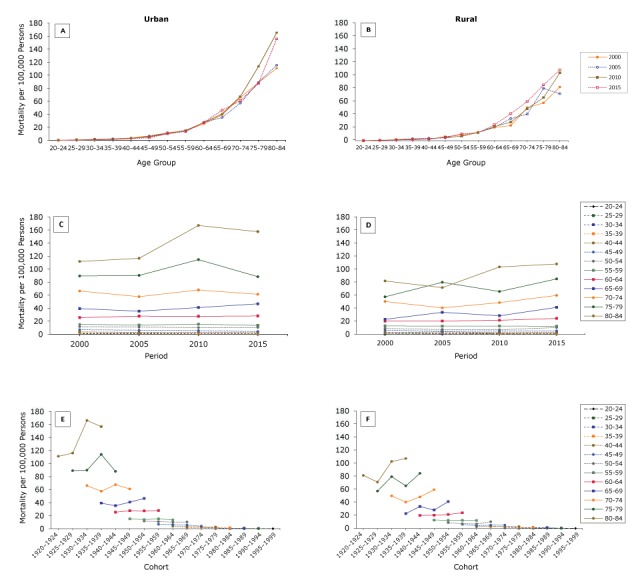
Differences in colorectal cancer mortality based on age, period, and cohort in urban and rural China, 2000–2015. A, Age-specific crude mortality of colorectal cancer by year in urban China, based on age. B, Age-specific crude mortality of colorectal cancer by year in rural China, based on age. C, Crude mortality of colorectal cancer in 13 age groups during 2000–2015 in urban China, based on period. D, Crude mortality of colorectal cancer in 13 age groups during 2000–2015 in rural China, based on period. E, Age-specific crude mortality of colorectal cancer in urban China, based on cohort. F, Age-specific crude mortality of colorectal cancer in rural China, based on cohort. Crude mortality rate (per 100,000 persons) based on data from *China Health Statistics Yearbook*. Data for 2000 were not available; thus, we used as a substitute the available data from the nearest year (2002).

CRC mortality increased with age in both rural and urban areas (Figure 2A, 2B). The rate accelerated beginning with the group aged 45 or older, and CRC mortality among people aged 55 or older in urban areas was higher than that of people in the same age group in rural areas. We found the highest CRC mortality among people aged 70 or older in 2010 in urban areas and 60 or older in 2015 in rural areas.

**Table:** 

Age Group	Figure 2A and 2C, Urban				Figure 2B and 2D, Rural			
	2000	2005	2010	2015	2000	2005	2010	2015
20-24	0.15	0.09	0.17	0.16	0.26	0.40	0.47	0.20
25-29	0.82	0.53	0.45	0.44	0.45	0.74	0.89	0.50
30-34	1.43	1.28	0.83	0.95	1.89	1.25	1.30	1.32
35-39	2.18	1.98	1.75	1.32	2.90	3.03	1.73	1.69
40-44	3.64	2.94	3.16	2.61	2.51	3.73	2.63	2.88
45-49	6.91	6.23	5.58	3.99	6.35	4.37	5.11	5.22
50-54	11.63	10.93	10.2	10.32	8.89	7.30	6.93	10.37
55-59	15.15	14.13	15.4	13.53	12.93	12.60	12.65	12.38
60-64	25.51	27.78	27.47	28.04	20.23	20.45	21.63	24.37
65-69	39.34	35.47	41.03	46.63	23.11	33.91	28.54	41.41
70-74	66.41	57.72	68.00	61.36	50.50	40.66	48.82	59.89
75-79	89.48	90.28	114.56	88.39	57.77	80.14	65.90	85.36
80-84	111.68	116.56	167.01	157.49	82.12	71.84	103.72	108.34

**Table:** 

Age	1920-24	1925-29	1930-34	1935-39	1940-44	1945-49	1950-54	1955-59	1960-64	1965-69	1970-74	1975-79	1980-84	1985-89	1990-94	1995-99
**Figure E, Urban**																
20-24	—	—	—	—	—	—	—	—	—	—	—	—	0.15	0.09	0.17	0.16
25-29	—	—	—	—	—	—	—	—	—	—	—	0.82	0.53	0.45	0.44	—
30-34	—	—	—	—	—	—	—	—	—	—	1.43	1.28	0.83	0.95	—	—
35-39	—	—	—	—	—	—	—	—	—	2.18	1.98	1.75	1.32	—	—	—
40-44	—	—	—	—	—	—	—	—	3.64	2.94	3.16	2.61	—	—	—	—
45-49	—	—	—	—	—	—	—	6.91	6.23	5.58	3.99	—	—	—	—	—
50-54	—	—	—	—	—	—	11.63	10.93	10.20	10.32	—	—	—	—	—	—
55-59	—	—	—	—	—	15.15	14.13	15.40	13.53	—	—	—	—	—	—	—
60-64	—	—	—	—	25.51	27.78	27.47	28.04	—	—	—	—	—	—	—	—
65-69	—	—	—	39.34	35.47	41.03	46.63	—	—	—	—	—	—	—	—	—
70-74	—	—	66.41	57.72	68.00	61.36	—	—	—	—	—	—	—	—	—	—
75-79	—	89.48	90.28	114.56	88.39	—	—	—	—	—	—	—	—	—	—	—
80-84	111.68	116.56	167.01	157.49	—	—	—	—	—	—	—	—	—	—	—	—
**Figure 2F, Rural**																
20-24	—	—	—	—	—	—	—	—	—	—	—	—	0.26	0.40	0.47	0.20
25-29	—	—	—	—	—	—	—	—	—	—	—	0.45	0.74	0.89	0.50	—
30-34	—	—	—	—	—	—	—	—	—	—	1.89	1.25	1.30	1.32	—	—
35-39	—	—	—	—	—	—	—	—	—	2.90	3.03	1.73	1.69	—	—	—
40-44	—	—	—	—	—	—	—	—	2.51	3.73	2.63	2.88	—	—	—	—
45-49	—	—	—	—	—	—	—	6.35	4.37	5.11	5.22	—	—	—	—	—
50-54	—	—	—	—	—	—	8.89	7.30	6.93	10.37	—	—	—	—	—	—
55-59	—	—	—	—	—	12.93	12.60	12.65	12.38	—	—	—	—	—	—	—
60-64	—	—	—	—	20.23	20.45	21.63	24.37	—	—	—	—	—	—	—	—
65-69	—	—	—	23.11	33.91	28.54	41.41	—	—	—	—	—	—	—	—	—
70-74	—	—	50.50	40.66	48.82	59.89	—	—	—	—	—	—	—	—	—	—
75-79	—	57.77	80.14	65.9	85.36	—	—	—	—	—	—	—	—	—	—	—
80-84	82.12	71.84	103.72	108.34	—	—	—	—	—	—	—	—	—	—	—	—

We found differences in trends between urban and rural areas among people aged 65 or older (Figure 2C, 2D). CRC mortality among people aged 70 or older in urban areas increased from 2005 to 2010 and then decreased from 2010 to 2015; we observed no change in CRC mortality among people younger than 70. In contrast, CRC mortality increased among people aged 65 or older in rural areas from 2005 to 2015 but did not change among people younger than 65.

We found differences in CRC mortality across cohort groups, and these differences reflected a significant urban–rural disparity (Figure 2E, 2F). CRC mortality among people aged 60 or older in urban areas increased at first and then decreased in more recent cohorts, whereas CRC mortality among people aged 60 or older in rural areas only increased across cohorts. We observed no obvious change in CRC mortality among people younger than 60.

We found significant age effects of CRC mortality in China, and increases in CRC mortality risk accelerated with age. The age effect of CRC mortality in urban areas was more obvious than in rural areas. After a natural exponential transformation, we found that relative risk of CRC mortality among people aged 60 to 64, compared with people aged 20 to 24, was 31.09 times larger in urban areas and 11.46 times larger in rural areas (Table 2).

**Table 2:** Age-Period-Cohort Analysis Results of Colorectal Cancer Mortality in Urban and Rural China, 2000–2015^a^

Effect	Urban			Rural		
	Coefficient (95% CI)	*P* Value^b^	Relative Risk	Coefficient (95% CI)	*P* Value^b^	Relative Risk
**Age**						
20–24	−2.69 (−5.3 to −0.02)	.05	1 [Reference]	−1.85 (−3.49 to −0.20)	.03	1 [Reference]
25–29	−1.52 (−2.81 to −0.24)	.02	3.19	−1.50 (−2.67 to −0.33)	.01	1.41
30–34	−1.08 (−2.12 to −0.05)	.04	4.95	−0.97 (−1.84 to 0.11)	.03	2.39
35–39	−0.92 (−1.83 to −0.02)	.05	5.82	−0.75 (−1.51 to 0.01)	.05	3.00
40–44	−0.67 (−1.45 to 0.10)	.09	7.48	−0.73 (−1.44 to −0.02)	.05	3.06
45–49	−0.31 (−0.96 to 0.34)	.36	10.79	−0.38 (−0.97 to 0.21)	.20	4.32
50–54	0.11 (−0.41 to 0.63)	.68	16.37	−0.10 (−0.59 to 0.39)	.68	5.72
55–59	0.25 (−0.16 to 0.67)	.23	18.90	0.21 (−0.18 to 0.61)	.29	7.84
60–64	0.75 (0.45 to 1.06)	<.001	31.09	0.59 (0.29 to 0.89)	<.001	11.46
65–69	1.03 (0.79 to 1.28)	<.001	41.10	0.89 (0.65 to 1.13)	<.001	15.48
70–74	1.37 (1.11 to 1.63)	<.001	57.65	1.25 (1.02 to 1.49)	<.001	22.17
75–79	1.69 (1.36 to 2.03)	<.001	79.48	1.58 (1.28 to 1.87)	<.001	30.69
80–84	1.99 (1.54 to 2.44)	<.001	107.16	1.76 (1.36 to 2.15)	<.001	36.70
**Period^c^**						
2000	−0.17 (−0.39 to 0.05)	.14	1 [Reference]	−0.21 (−0.42 to −0.01)	.04	1 [Reference]
2005	−0.15 (−0.27 to −0.04)	.01	1.01	−0.10 (−0.22 to 0.02)	.10	1.12
2010	0.14 (0.03 to 0.25)	.01	1.36	0 (−0.12 to 0.12)	.97	1.24
2015	0.18 (−0.03 to 0.40)	.10	1.42	0.31 (0.12 to 0.51)	.002	1.69
**Cohort**						
1920–1924	0.89 (0.29 to 1.50)	.004	1 [Reference]	0.93 (0.37 to 1.49)	.001	1 [Reference]
1925–1929	0.94 (0.46 to 1.43)	<.001	1.05	0.72 (0.27 to 1.16)	.002	0.81
1930–1934	0.99 (0.60 to 1.37)	<.001	1.09	0.96 (0.60 to 1.32)	<.001	1.03
1935–1939	0.88 (0.55 to 1.21)	<.001	0.99	0.65 (0.34 to 0.96)	<.001	0.76
1940–1944	0.66 (0.32 to 1.00)	<.001	0.79	0.68 (0.37 to 0.99)	<.001	0.78
1945–1949	0.60 (0.20 to 0.99)	.003	0.74	0.58 (0.21 to 0.95)	.002	0.70
1950–1954	0.54 (0.06 to 1.03)	.03	0.70	0.56 (0.11 to 1.01)	.02	0.69
1955–1959	0.39 (−0.21 to 0.99)	.20	0.61	0.37 (−0.18 to 0.92)	.19	0.57
1960–1964	0.16 (−0.57 to 0.88)	.67	0.48	0.05 (−0.62 to 0.72)	.88	0.41
1965–1969	−0.04 (−0.87 to 0.79)	.93	0.39	0.16 (−0.57 to 0.89)	.68	0.46
1970–1974	−0.38 (−1.34 to 0.58)	.43	0.28	−0.15 (−0.97 to 0.67)	.72	0.34
1975–1979	−0.57 (−1.61 to 0.48)	.29	0.23	−0.60 (−1.54 to 0.35)	.22	0.22
1980–1984	−1.06 (−2.33 to 0.20)	.10	0.14	−0.85 (−1.89 to 0.18)	.11	0.17
1985–1989	−1.26 (−2.84 to 0.32)	.12	0.12	−0.85 (−2.06 to 0.36)	.17	0.17
1990–1994	−1.41 (−3.70 to 0.88)	.23	0.10	−1.19 (−3.04 to 0.66)	.21	0.12
1995–1999	−1.33 (−6.57 to 3.91)	.62	0.11	−2.01 (−6.38 to 2.36)	.37	0.05

Abbreviation: CI, confidence interval.

a Data source: *China Health Statistics Yearbook* (15).

b Determined by *z* test.

c Data for 2000 were not available; thus, we used as a substitute the available data from the nearest year (2002).

The relative risk of CRC mortality increased with period from 2000 to 2015 nationally, and we observed a significant urban–rural disparity (Table 2). The relative risk of CRC mortality accelerated mostly from period 2005 to period 2010 in urban China, whereas relative risk accelerated mostly from period 2010 to period 2015 in rural China. Compared with period 2000, the relative risk of CRC mortality in urban areas was 1.01 in period 2005 (vs 1.12 in rural areas), 1.36 in period 2010 (vs 1.24 in rural areas), and 1.42 in period 2015 (vs 1.69 in rural areas).

CRC mortality risk was lower among more recent cohorts than among earlier cohorts (Table 2). Compared with people who were born from 1920 to 1924, the relative risk of CRC mortality among people born from 1950 to 1954 was 0.70 times smaller in urban areas and 0.69 times smaller in rural areas.

## Discussion

The trend in CRC mortality increased from 2000 to 2015 in both urban and rural areas of China, which is consistent with findings in other developing countries such as some South American countries, Romania, and Russia (3). However, the trend in CRC mortality appears to be decreasing in developed countries (3). For example, in the United States, the annual percentage change in CRC mortality was −2.5% from 2005 to 2015 (2), and in Austria, it was −1.8% among men and −2.4% among women from 2002 to 2011 (3). The decline in CRC mortality in developed countries can be largely attributed to improvements in CRC screening technology and screening strategies. Many studies showed that the use of fecal occult blood testing in mass screening was associated with a modest reduction in CRC mortality, resulting in early detection and a 16% overall reduction in CRC mortality (18). The results of one study suggested a 40% to 60% lower risk of incident colorectal cancer and death from CRC after screening colonoscopy (significant only for deaths of proximal colon cancer) (19). China is one of the largest developing countries, and it is going through a rapid economic growth and medical reform. The diet of people in China and other factors related to CRC are also changing rapidly. Thus, it is necessary to identify CRC mortality trends from various temporal perspectives in China.

Older age is a risk factor for CRC mortality in both urban and rural areas of China. The relative risk of people aged 60 to 64, compared with people aged 20 to 24, was 31.09 times larger in urban areas and 11.46 times larger in rural areas. A study in rural China showed that the risk of benign tumors in people aged 75 to 79 was 38 times higher than the risk in people aged 5 to 9 (12). Studies on CRC in Taiwan and Serbia showed that aging increased the risk of CRC mortality (13,20). Many studies also showed that most CRC deaths occurred among people aged 50 years or older (21). Somatic function usually declines with aging, and CRC risk factors also accumulate with aging (12).

CRC mortality increased gradually from 2000 to 2015 in China. This increase might be caused by the increase in “westernization” risk factors. Since the implementation of reform and opening-up policy, the living environment and lifestyle of Chinese residents have westernized rapidly. A few studies from China showed that personal risk behaviors, such as insufficient physical activity, are becoming increasingly common and that diet has changed into a high-fat, low-fiber diet (7,18). For example, from 1990 to 2009, the physical activity of people living in low-density urban areas decreased from 500 metabolic-equivalent (MET)-hours per week to 300 MET-hours per week and in high-density urban areas decreased from 200 MET-hours per week to 125 MET-hours per week (18). From 2000 to 2012, obesity prevalence in China increased from 7.1% to 11.9%, fat intake increased from 76.3 grams per day to 79.9 grams per day, and dietary fiber decreased from 12.0 grams per day to 10.8 grams per day (7). Some studies, including a study in China, showed that smoking, insufficient physical activity, and insufficient vegetable and fruit intake could increase CRC mortality (4,23).

The relative risk of period effect in urban areas was higher than that in rural areas before period 2010. However, the relative risk in rural areas increased quickly and exceeded the relative risk of period effect in urban areas in period 2015. Inequitable distribution of health care services between urban and rural areas may explain the differences in period effect. Compared with rural residents, urban residents have better health care services (24). The numbers of health workers, licensed physicians, and registered nurses per 1,000 people in urban areas of China was 2.2 times, 2.0 times, and 2.7 times larger, respectively, than numbers in rural areas in 2015 (15). In addition, the average annual funding for basic medical insurance per urban worker was approximately 2,500 Chinese yuan (in US dollars, approximately $400), which was 5 times higher than the average annual funding per rural resident in 2015 (15). On the other hand, pilot CRC screening strategies were put into place by the Chinese government in 2012 (11,25). However, these programs were conducted only in urban areas; rural areas generally lack adequate field conditions, implementation funding, and screening equipment (26).

We observed lower CRC mortality in more recent cohorts, a finding that is consistent with previous research (12). Several major events occurred in China during 1920–1990, such as World War II (1939–1945) and the Great Famine (1959–1961), when food and health care services, especially for pregnant women and infants, could not meet demand (27). Nutritional status in early childhood affects biological processes at older ages, including the susceptibility of the colorectal epithelium to malignant transformation (28,29), and can lead to chronic diseases. However, the cohort effect observed in our study contradicts evidence in some other studies (30,31). This contradiction may be explained by differences in race, national history, and anatomic subsites of CRC. A study on CRC mortality in Nordic countries and Estonia showed that the relative risk of CRC mortality was higher in a more recent cohort (30). A study on the CRC incidence rate in Sweden showed that different anatomic subsites of CRC had different cohort effects (31). Compared with people born in 1925, people born in more recent cohorts had a higher relative risk of right-sided colon cancer and rectal cancer but a lower relative risk of left-sided colon cancer (31).

A study by Arnold et al showed that both CRC incidence and mortality increased in China from 1990 to 2000 (3). The Global Burden of Disease Study 2016 showed that CRC incidence in China increased from 13.05 per 100,000 persons in 2000 to 22.76 per 100,000 persons in 2011 (32), and a study by Chen et al showed that the average annual percentage change in CRC incidence in China was 4.2% from 2000 to 2011 and 1.3% from 2006 to 2012 (33). The consistent increase in CRC incidence and mortality reflects the severity of the burden of CRC in China. However, the Cancer Early Detection and Treatment Project is the only national screening strategy in China. The project is not yet ready to be implemented beyond trials in a few major cities, and it is restricted to people aged 40 to 69 in urban China (34). Although a large percentage (40.8%) of the Chinese population was aged 40 to 69 in 2016, 6.7% of the population was aged 70 or older and 42.6% of the population was living in rural China (35). Thus, most Chinese residents do not qualify for this program. According to our study, interventions such as national screening programs in both urban and rural areas, especially among people aged 60 or older, are needed to reduce the burden of CRC in China.

This study has several limitations. First, no data on age-specific CRC incidence were available from 2000 to 2015 in China; this lack of data limited our ability to explain the trend of CRC incidence. A comprehensive analysis on CRC incidence should be conducted. Additionally, because we had no reliable data on anatomic subsite and our knowledge about the comprehensive effects of the risk factors of CRC was limited, some explanation of the APC models should be interpreted with caution. Furthermore, a longer study period may enable us to gain a better understanding of mortality trends. However, only data during 2000–2015 (4 time points in the intrinsic estimator method) were available, even when we replaced mortality data in 2000 with mortality data in 2002.

We found that CRC mortality in urban areas of China was higher than CRC mortality in rural areas and that the annual average percentage change in CRC mortality was higher in urban areas. The APC analysis showed that the CRC mortality increased with age and period but decreased with cohort. In China, more interventions to reduce the burden of CRC should be conducted, and it is more necessary for older people and urban residents to adopt a healthy lifestyle. 

## References

[1] Ferlay J , Soerjomataram I , Dikshit R , Eser S , Mathers C , Rebelo M , Cancer incidence and mortality worldwide: sources, methods and major patterns in GLOBOCAN 2012. Int J Cancer 2015;136(5):E359–86. 10.1002/ijc.29210 25220842

[2] Siegel RL , Miller KD , Fedewa SA , Ahnen DJ , Meester RGS , Barzi A , Colorectal cancer statistics, 2017. CA Cancer J Clin 2017;67(3):177–93. 10.3322/caac.21395 28248415

[3] Arnold M , Sierra MS , Laversanne M , Soerjomataram I , Jemal A , Bray F . Global patterns and trends in colorectal cancer incidence and mortality. Gut 2017;66(4):683–91. 10.1136/gutjnl-2015-310912 26818619

[4] Johnson CM , Wei C , Ensor JE , Smolenski DJ , Amos CI , Levin B , Meta-analyses of colorectal cancer risk factors. Cancer Causes Control 2013;24(6):1207–22. 10.1007/s10552-013-0201-5 23563998 PMC4161278

[5] Edwards BK , Ward E , Kohler BA , Eheman C , Zauber AG , Anderson RN , Annual report to the nation on the status of cancer, 1975–2006, featuring colorectal cancer trends and impact of interventions (risk factors, screening, and treatment) to reduce future rates. Cancer 2010;116(3):544–73. 10.1002/cncr.24760 19998273 PMC3619726

[6] Popkin BM . The nutrition transition in low-income countries: an emerging crisis. Nutr Rev 1994;52(9):285–98. 10.1111/j.1753-4887.1994.tb01460.x 7984344

[7] National Health Commission of the People’s Republic of China. Report on Chinese residents’ chronic diseases and nutrition, 2015. Beijing (CN): People’s Medical Publishing House; 2015. p. 77–103.

[8] Huang K , Song YT , He YH , Feng XL . Health system strengthening and hypertension management in China. Glob Health Res Policy 2016;1(1):13. 10.1186/s41256-016-0013-8 29202062 PMC5693514

[9] Su L , Sun L , Xu L . Review on the prevalence, risk factors and disease management of hypertension among floating population in China during 1990–2016. Glob Health Res Policy 2018;3(1):24. 10.1186/s41256-018-0076-9 30123839 PMC6091177

[10] Lv L , Deng S . The current status and development strategies of chronic disease management in China. Chinese Journal of Health Policy 2016;7:1–7.

[11] Chen XZ , Hu JK , Zhou ZG . Importance of organized screening and surveillance for colorectal cancer in China: epidemiological differences from Europe. Eur J Cancer Prev 2015;24(6):459–60. 10.1097/CEJ.0000000000000105 26398321

[12] Wang P , Xu C , Yu C . Age-period-cohort analysis on the cancer mortality in rural China: 1990–2010. Int J Equity Health 2014;13(1):1. 10.1186/1475-9276-13-1 24383432 PMC4029464

[13] Su SY , Huang JY , Jian ZH , Ho CC , Lung CC , Liaw YP . Mortality of colorectal cancer in Taiwan, 1971–2010: temporal changes and age-period-cohort analysis. Int J Colorectal Dis 2012;27(12):1665–72. 10.1007/s00384-012-1521-8 22772747

[14] World Health Organization. International statistical classification of diseases and related health problems, 10th revision. Geneva (CH): World Health Organization; 1992.

[15] National Health Commission of the People’s Republic of China. China Health Statistics Yearbook. Beijing (CN): Peking Union Medical College Press; 2002–2016. p. 284–306.

[16] Kim HJ , Fay MP , Feuer EJ , Midthune DN . Permutation tests for joinpoint regression with applications to cancer rates. Stat Med 2000;19(3):335–51. 10.1002/(SICI)1097-0258(20000215)19:3<335::AID-SIM336>3.0.CO;2-Z 10649300

[17] Yang Y , Schulhofer-Wohl S , Fu WJ , Land KC . The intrinsic estimator for age-period-cohort analysis: what it is and how to use it. Am J Sociol 2008;113(6):1697–736. 10.1086/587154

[18] Zavoral M , Suchanek S , Majek O , Fric P , Minarikova P , Minarik M , Colorectal cancer screening: 20 years of development and recent progress. World J Gastroenterol 2014;20(14):3825–34. 10.3748/wjg.v20.i14.3825 24744575 PMC3983439

[19] Brenner H , Stock C , Hoffmeister M . Effect of screening sigmoidoscopy and screening colonoscopy on colorectal cancer incidence and mortality: systematic review and meta-analysis of randomised controlled trials and observational studies. BMJ 2014;348:g2467. 10.1136/bmj.g2467 24922745 PMC3980789

[20] Ilic M , Ilic I . Colorectal cancer mortality trends in Serbia during 1991–2010: an age-period-cohort analysis and a joinpoint regression analysis. Chin J Cancer 2016;35(1):55. 10.1186/s40880-016-0118-y 27333993 PMC4918103

[21] Attard SM , Howard AG , Herring AH , Zhang B , Du S , Aiello AE , Differential associations of urbanicity and income with physical activity in adults in urbanizing China: findings from the population-based China Health and Nutrition Survey 1991–2009. Int J Behav Nutr Phys Act 2015;12(1):152. 10.1186/s12966-015-0321-2 26653097 PMC4676871

[22] Bosetti C , Levi F , Rosato V , Bertuccio P , Lucchini F , Negri E , Recent trends in colorectal cancer mortality in Europe. Int J Cancer 2011;129(1):180–91. 10.1002/ijc.25653 20824701

[23] Murphy G , Shu XO , Gao YT , Ji BT , Cook MB , Yang G , Family cancer history affecting risk of colorectal cancer in a prospective cohort of Chinese women. Cancer Causes Control 2009;20(8):1517–21. 10.1007/s10552-009-9353-8 19418234 PMC2843785

[24] Anand S , Fan VY , Zhang J , Zhang L , Ke Y , Dong Z , China’s human resources for health: quantity, quality, and distribution. Lancet 2008;372(9651):1774–81. 10.1016/S0140-6736(08)61363-X 18930528

[25] Dong Z , Qiao Y . The practice and discussion of Population-based Cancer Screening Program in China. China Cancer 2009;9:686–9.

[26] Zheng S , Zhang S , Cai Z . Protocol and practice for colorectal cancer screening. China Cancer 2009;18:700–4.

[27] Hesketh T , Wei XZ . Health in China. From Mao to market reform. BMJ 1997;314(7093):1543–5. 10.1136/bmj.314.7093.1543 9183206 PMC2126766

[28] Abu-Saad K , Fraser D . Maternal nutrition and birth outcomes. Epidemiol Rev 2010;32(1):5–25. 10.1093/epirev/mxq001 20237078

[29] Waterland RA , Jirtle RL . Early nutrition, epigenetic changes at transposons and imprinted genes, and enhanced susceptibility to adult chronic diseases. Nutrition 2004;20(1):63–8. 10.1016/j.nut.2003.09.011 14698016

[30] Svensson E , Møller B , Tretli S , Barlow L , Engholm G , Pukkala E , Early life events and later risk of colorectal cancer: age-period-cohort modelling in the Nordic countries and Estonia. Cancer Causes Control 2005;16(3):215–23. 10.1007/s10552-004-3073-x 15947873

[31] Thörn M , Bergström R , Kressner U , Sparén P , Zack M , Ekbom A . Trends in colorectal cancer incidence in Sweden 1959–93 by gender, localization, time period, and birth cohort. Cancer Causes Control 1998;9(2):145–52. 10.1023/A:1008826109697 9578291

[32] Institute for Health Metrics and Evaluation. GBD Compare Viz Hub. https://vizhub.healthdata.org/gbd-compare/. Accessed November 1, 2016.

[33] Chen W , Zheng R , Baade PD , Zhang S , Zeng H , Bray F , Cancer statistics in China, 2015. CA Cancer J Clin 2016;66(2):115–32. 10.3322/caac.21338 26808342

[34] Ministry of Health of the People’s Republic of China . Administrative measures for the cancer early detection and treatment project in urban areas [in Chinese]. http://www.nhfpc.gov.cn/jkj/s5878/201211/15cc2ee847bc46e6b3c0ffe3fb4f159a.shtml. Accessed November 1, 2012.

[35] National Bureau of Statistics of the People’s Republic of China. China statistical yearbook. Beijing (CN): China Statistics Press; 2017. p. 32.

